# Reduced risk of all-cancer and solid cancer in Taiwanese patients with rheumatoid arthritis treated with etanercept, a TNF-α inhibitor

**DOI:** 10.1097/MD.0000000000006055

**Published:** 2017-02-17

**Authors:** Joung-Liang Lan, Chun-Hung Tseng, Jiunn-Horng Chen, Chi-Fung Cheng, Wen-Miin Liang, Gregory J. Tsay

**Affiliations:** a School of Medicine, China Medical University; b Division of Immunology and Rheumatology, Department of Internal Medicine, China Medical University Hospital; c Rheumatology Research Laboratory, China Medical University; d Department of Neurology, China Medical University Hospital; e Graduate Institute of Biostatistics, Biostatistics Center; f Department of Public Health, China Medical University, Taichung, Taiwan.

**Keywords:** all-cancer, etanercept, rheumatoid arthritis, solid cancer, TNF-α inhibitor

## Abstract

Biologics has been widely used in the treatment of rheumatoid arthritis. We aimed to determine whether etanercept, a TNF-α inhibitor (TNFi) that is used to treat patients with rheumatoid arthritis (RA), affects cancer risk.

This retrospective matched cohort study used data in the Registry of Catastrophic Illness Database in Taiwan from January 1, 1996 to December 31, 2010. RA, all-cancer, and solid cancer were defined using International Classification of Disease codes (ICD-9-CM 714.X, 140–208, and 140–199, respectively). Cox proportional hazard modeling was used to estimate the hazard ratio (HR) of cancer in all TNFi-treated RA patients, with a focus on the risk in the etanercept-treated patients, after adjusting for comorbidities and concomitant medication.

In this Taiwanese dataset, there were 1111 TNFi-treated RA patients and 16,812 RA patients who were naive to all biologics identified. Among the 1002 pairs of etanercept-treated and biologic-naive patients who were matched 1-to-1 for age, gender, RA duration, methotrexate-use, and index date of TNFi prescription, the mean age was 48.9 ± 15.0 years. The highest proportion of patients was in the age subgroup of 30 to 60 years (63.8%). Most patients (77.2%) were women. The mean RA duration before etanercept treatment was 2.0 ± 1.5 years. During a mean 2.1 years of observation, etanercept was associated with significant risk reduction for all-cancer (HR 0.59, 0.36–0.98) and solid cancer (HR 0.46, 0.27–0.79) relative to the matched biologic-naive patients.

The current study explored the safety profile of TNFi and identified a potential benefit of etanercept on the incidence of all-cancer and solid cancer in RA patients.

## Abstract 2

Supplemental Digital Content is available in the text

## 1 Introduction

Epidemiologic studies have reported a higher risk of cancer in patients with rheumatoid arthritis (RA) relative to the general population.^[1–4]^ Most attention has been devoted to the risk of hematological malignancy in RA patients.^[5,6]^ But the more common solid cancers have attracted recent attention.^[7]^ The incidence of lung cancer^[1–3]^ and cervical cancer^[4]^ is higher in RA patients than in the general population, and the incidence of breast and colon cancers is lower.^[2,3,6,7]^ A higher risk of kidney and genital cancers has also been reported among RA patients in Taiwan.^[1]^

It is still questionable whether RA treatment with synthetic disease-modifying antirheumatic drugs (DMARDs) increases the risk of cancer.^[8–11]^ Although TNF-α is one of the proinflammatory cytokines involved in the chronic inflammation in RA,^[12,13]^ accumulating evidence indicates a complex role of TNF-α in the development and progression of malignancy.^[12,14]^ In the era of treatment with TNF-α inhibitors (TNFi), concerns have been raised about impaired immunity with an increased potential risk of infections and malignancies. Evidence regarding an association between cancer occurrence and the use of TNFi is inconclusive.^[15,16]^

Some studies have reported a higher incidence of malignancy in RA patients treated with TNFi than in RA patients treated with traditional DMARDs.^[16–20]^ The increased risk of cancer included hematologic malignancy^[17,18]^ and nonmelanoma skin cancer.^[18,19]^ However, recent observational studies and meta-analyses indicated no overall increased risk of malignancy except skin cancer.^[5–7,19,21–24]^ Use of TNFi in patients with a history of breast cancer or carcinoma in situ of the cervix did not increase the recurrence rate.^[25,26]^ Furthermore, a potential effect of TNFi in suppressing tumor progression by disrupting TNF-α-related signaling with respect to tumor-promoting inflammation has been reported.^[27–29]^

This study aimed to investigate the impact of treatment with TNFi, especially etanercept, a popular TNFi in Taiwan, on the development of all-cancer and solid cancer in RA patients.

## 2 Materials and methods

### 2.1 Data source

This was a retrospective, population-based cohort study performed using the claims database from the National Health Insurance Research Database (NHIRD) in Taiwan from January 1, 1996 to December 31, 2010. The National Health Insurance (NHI) program in Taiwan, which started in 1995, covers >99% of the national population.^[1]^ The NHIRD thus provides reliable information with which to explore the risk of malignancy development in RA patients.^[1]^ The ethical review board of the China Medical University in Taiwan approved this study (DMR101-IRB1-138).

### 2.2 Definition of the RA patients

The International Classification of Diseases, 9th Revision, Clinical Modification (ICD-9-CM) code was used for coding the diseases of interest in the present study. The database of RA patients was compiled from the Registry of Catastrophic Illness Database, a subsection of the NHIRD.^[1]^ This database tracks patients in the NHI registry system with catastrophic illnesses, including autoimmune diseases such as RA, systemic lupus erythematosus, systemic sclerosis, Sjögren syndrome, and cancer. The Bureau of NHI requires each diagnosis of catastrophic illness to be confirmed by at least 2 specialists who carefully review original medical records, laboratory data, and imaging findings. To be eligible for a cancer catastrophic illness certificate, patients must further provide cytological or pathological reports.

RA patients included in this study were diagnosed with RA (ICD-9-CM 714.X) for the first time between January 1, 1999 and December 31, 2008. Those who received treatment with TNFi, such as etanercept and adalimumab, were defined as the TNFi-treated group. Infliximab and certolizumab are currently not available in the Taiwanese market, neither was golimumab available in Taiwan until 2012. The NHI guidelines restrict the prescription of TNFi for RA patients with active disease (defined as DAS28 score >5.1) after treatment with a full dose of methotrexate (MTX) and one other DMARD for >6 months in total. The patients (either TNFi-treated or biologic-naive) were considered to have used one of these DMARDs if they had 1 inpatient claim or 3 outpatient claims in the past 6 months before the index date (1st day of TNFi use). The use of DMARDs, including MTX, hydroxychloroquine, sulfasalazine, and leflunomide, was determined using the claims record for each patient. The use of biologics is reimbursed by the NHI program for RA patients who do not have latent tuberculosis and are not chronic hepatitis B or C carriers. Those infected patients of either latent tuberculosis or chronic hepatitis are requested to receive a complete course of treatment before reimbursement for biologics use. However, those with solid organ transplants, or those with HIV or HPV infection are not regulated or monitored in the NHI program.

The reference patients were selected from the RA patients who were naive to all biologics including TNFi and rituximab during the entire observation period. The index date for each TNFi-treated patient was assigned as the 1st day of TNFi use, and for the biologic-naive group the index dates were randomly assigned to match with the TNFi-treated group. These patients were followed up until malignancy occurrence, death, dropout from the NHI program, or December 31, 2010. Each subject was followed up for a mean of 3.9 years (Table 1) and a maximum of 12 years (from 2008 to 2010).

**Table 1:**
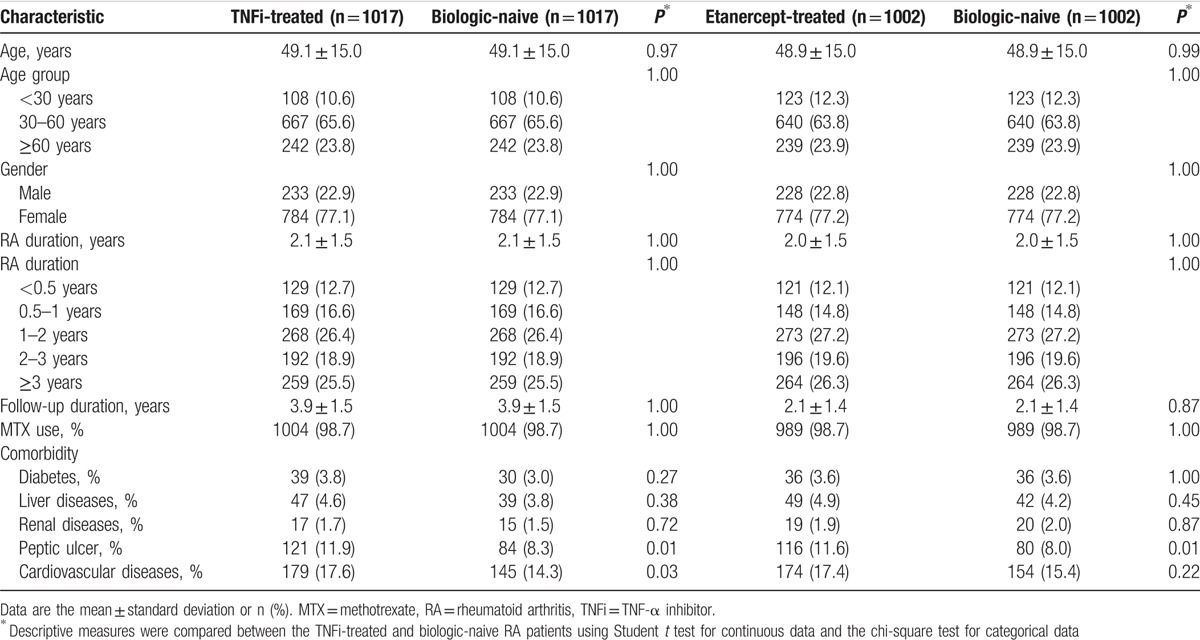
Demographics of RA patients treated with TNFi or etanercept and the respective case-matched RA patients who were naive to all biologics.

Comorbidities included cardiovascular diseases (ICD-9-CM 390–459), gastrointestinal disease (ICD-9-CM 530–533, 535–536), renal dysfunction (ICD-9-CM 580–586), diabetes (ICD-9-CM 250), and liver diseases (ICD-9-CM 571–573).

### 2.3 Outcomes

**Figure 1:**
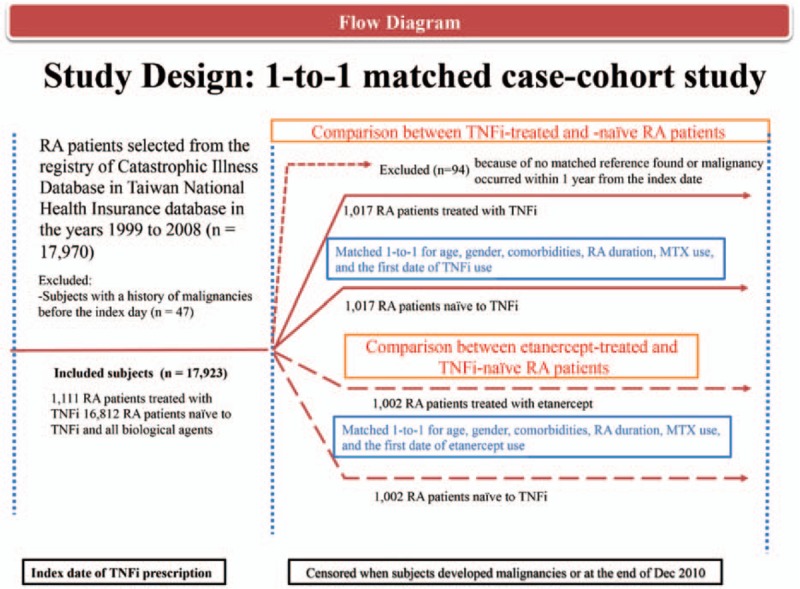
Flow diagram of the study design.

The primary outcome was all-cancer (ICD-9-CM 140–208), and the secondary outcome was solid cancer (ICD-9-CM 140–199). These diagnoses were identified based on the records from the Registry of Catastrophic Illness Database. Enrolled subjects with a history of malignancy before the index date were excluded (n = 47; Fig. 1, Supplementary Table 1). We did not include patients with in situ malignancies (ICD-9-CM 230–234) because these do not qualify for a catastrophic illness certificate. Solid cancers comprised all-cancers except hematologic malignancy (ICD-9-CM 200–208). Skin cancers (ICD-9-CM 172–173) were not included in the definition of solid cancers for this study.

### 2.4 One-to-one matching scheme between TNFi-treated and biologic-naive RA patients

The TNFi-treated patients comprised RA patients who received etanercept or adalimumab. The biologic-naive RA patients were matched 1-to-1 with the TNFi-treated patients by age, sex, RA duration, MTX use, and index date of TNFi prescription. Due to a difficulty in matching multiple covariates at baseline between TNFi-treated and biologic-naive RA patients, 1-to-1 matching scheme was chosen instead of one-to-multiple matching. Those TNFi-treated RA patients must have at least 3 cumulative doses of drug survival without immediate adverse effect. Those who could not tolerate TNFi may have been switched to other biologics and were excluded from the current study. To prevent misclassification of preexisting cancer as incident cancer, enrolled TNFi-treated subjects with malignancy occurrence within 1 year from the index date (n = 3) and their matched biologic-naive comparators (n = 3) were excluded.

Using a similar matching technique in separate analyses, subgroup analyses were performed to assess the impact of different TNFi on the development of malignancy. Etanercept has been in the Taiwanese market since 2003, but adalimumab was not available until 2007. There were few 1st-time users of adalimumab during the study period, and any etanercept-treated patients who were switched to adalimumab or rituximab were excluded (n = 109 for adalimumab users, n = 47 for rituximab users). Only the subgroup treated with etanercept alone (n = 1002) had sufficient power for analysis.

### 2.5 Statistical analysis

Demographic data were compared between TNFi-treated and biologic-naive RA patients. Incidence rates of all-cause cancers and solid cancers were calculated and compared between TNFi-treated and the matched biologic-naive RA patients using the Wald test from the Poisson model. Stratified Cox proportional hazard modeling was used to estimate the adjusted hazard ratio (aHR) of cancer in patients treated with TNFi and in those treated with etanercept only. The potential confounders were chosen by a stepwise selection model and included age, gender, comorbidities, RA duration, cumulative dose of TNFi, and the cumulative dose of MTX and/or other DMARDs (assessed for those prescribed for at least a 30-day period) and nonsteroid antiinflammatory drugs (assessed for those prescribed for at least a 30-day period). Subgroup analysis in the matched pairs was performed with respect to age (<30 years, 30–60 years, and ≥60 years), gender, and RA duration (<1 year, 1–2 years, and ≥2 years). SAS software (version 9.2; SAS Institute, Cary, NC) was used for the data analysis.

## 3 Results

### 3.1 SIR of all-cancer and solid cancer

There were 1111 RA patients treated with TNFi and 16,812 patients naive to all biologics identified from the Registry of Catastrophic Illness Database (Fig. 1). Malignancy occurrence was expressed as a standardized incidence ratio (SIR) for studied patients during the observation period relative to the age-specific and gender-specific incidence rate obtained from the World Health Organization.^[30]^ Preliminarily, we found the SIR of all-cancer relative to the general population was 0.80 (95% confidence interval [CI] 0.54–1.15) in RA patients treated with TNFi and 1.33 (95% CI 1.23–1.43) in patients who were naive to biologics (no tabulated data presented).

### 3.2 Demographic data

Among 1111 RA patients treated with TNFi, 94 patients did not match with a reference according to the matching criteria or diagnosed as a malignancy in the 1st year of follow-up in this analysis. A total of 1017 pairs of TNFi-treated and biologic-naive RA patients matched for age, gender, RA duration, MTX use, and index date of TNFi prescription were compared (Table 1). A very high proportion of MTX use (98.7%) in both TNFi-treated and biologic-naive patients was consistent with the NHI guidelines, which suggest a full dose of MTX use before application of biologics. Those (1.3%) who could not tolerate MTX were switch to other DMARD.

The demographic data of 1002 etanercept-treated RA patients and 1002 matched biologic-naive patients were also analyzed. The mean age of the etanercept-treated patients was 48.9 ± 15.0 years. The highest proportion of patients was in the age subgroup of 30 to 60 years (63.8%). Most patients (77.2%) were women. Other than peptic ulcer, the prevalence of comorbidities was not significantly different between the etanercept-treated and the matched biologic-naive cohorts.

The mean disease duration of RA before the index date for RA patients who received etanercept was 2.0 ± 1.5 years (Table 1), and the mean duration of etanercept use was 2.26 ± 1.31 years (Table 2). The proportion of patients using each medication after the index date, including MTX, other DMARDs (hydroxychloroquine, leflunomide, and sulfasalazine), and corticosteroid, and the duration of drug use were significantly higher in the etanercept-treated patients than in the biologic-naive patients (Table 2). The greater use of drugs and the longer duration of drug use suggest that the etanercept-treated patients had a more severe inflammatory condition than the biologic-naive patients, which agree with the required practice procedures of the NHI that RA patients treated with TNFi should be those individuals with active inflammatory arthritis after full treatment with MTX and a 2nd DMARD.

**Table 2:**
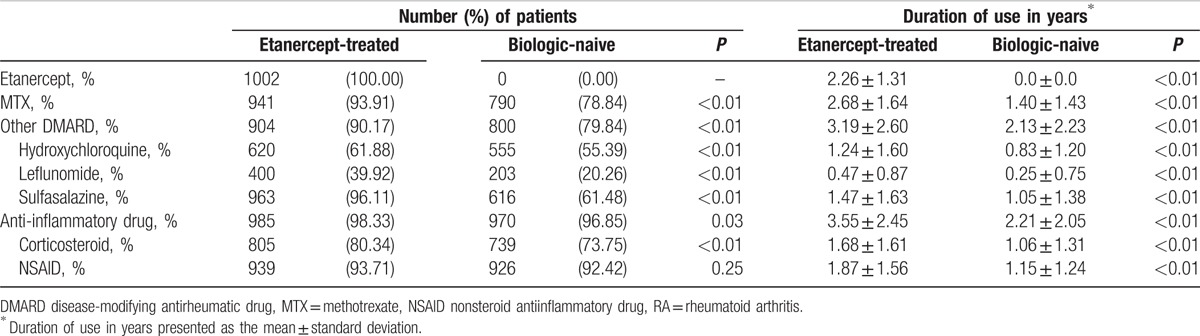
Medication use after the index date in the etanercept-treated and the matched biologic-naive RA patients.

### 3.3 Association of etanercept use with the development of malignancy in RA patients

**Figure 2:**
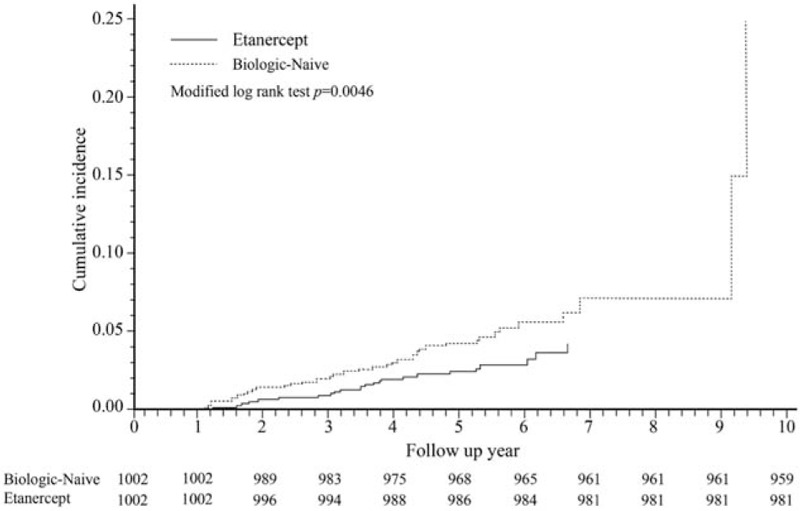
Cumulative incidence of all-cancer in the etanercept-treated rheumatoid arthritis (RA) patients and the 1-to-1 matched biologic-naive RA patients. Etanercept-treated patients who had a cancer occurrence during their 1st year of observation (n = 3) and their matched references were excluded.

Between the matched TNFi-treated and biologic-naive RA patients, the overall mortality was comparable, with 77 deaths in the TNFi-treated cohort and 72 deaths in the biologic-naive cohort (7.6% and 7.1%, respectively). The difference in the cumulative incidence of all-cancer in the etanercept-treated and the matched biologic-naive patients was assessed and plotted using Gray test on the basis of Fine and Gray model,^[2]^ that is, the subdistribution proportional hazards model, with death considered as the competing risk (Fig. 2). RA patients treated with etanercept had a significantly lower incidence rate of all-cancer than RA patients who were naive to biologics.

Table 3 shows that the incidence rates of all-cancer and solid cancer in the etanercept-treated patients were both lower than those in the matched biologic-naive patients (IRR 0.58, *P* = 0.03 and IRR 0.47, *P* < 0.01, respectively). In the subgroup analysis by age, there was a significantly lower occurrence of all-cancer and solid cancer in patients treated with etanercept who were ≥60 years old. For both male and female RA patients, those treated with etanercept had a significantly lower occurrence of solid cancer. For RA disease duration of ≥1 year, the occurrence of solid cancer in patients treated with etanercept was significantly lower than that of biologic-naive patients.

**Table 3:**
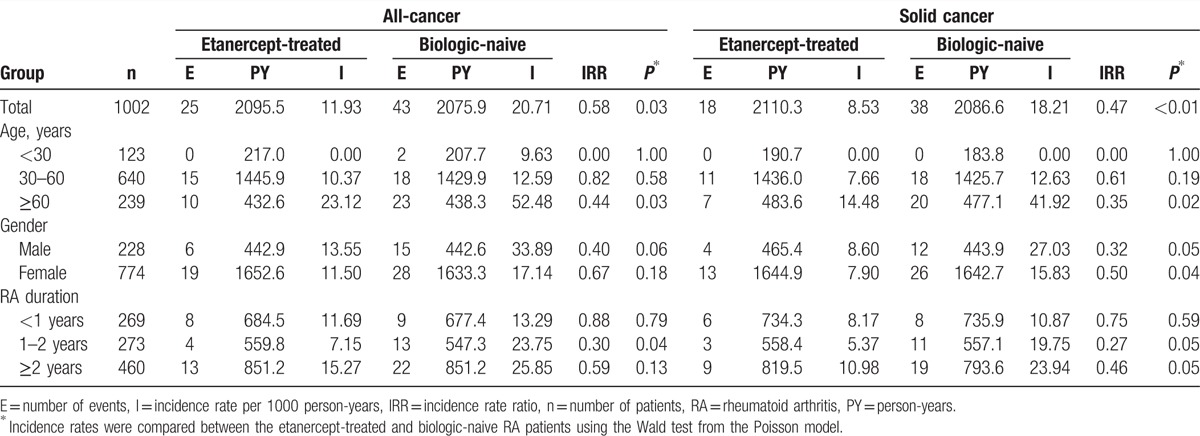
Incidence rate of all-cancer and solid cancer in the 1002 case-matched etanercept-treated and biologic-naive RA patients.

Cox proportional hazard modeling revealed a significant reduction in the risk of all-cancer in the etanercept-treated patients (aHR 0.59, 95% CI 0.36–0.98) after adjusting covariates in a stepwise selection model (Table 4). Similarly, there was a significant reduction in the risk of solid cancer (aHR 0.46, 95% CI 0.27–0.79) relative to the biologic-naive patients.

**Table 4:**
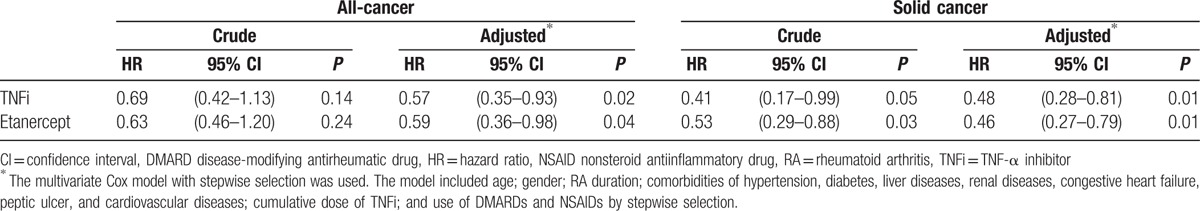
Cox proportional hazard model analysis for risk of all-cancer and solid cancer in TNFi-treated RA patients relative to the case-matched TNFi-naive RA patients.

## 4 Discussion

Compatible with previous reports, our findings confirmed the potential benefit of TNFi on the incidence of malignancy that was previously reported in Taiwanese^[31]^ and British^[32]^ patients. Etanercept, the 1st approved and most commonly used TNFi in Taiwan during the study period, was associated with a significant reduction in risk of all-cancer and solid cancer.

Recent studies, including meta-analyses, suggest that there is no increase in cancer risk in RA patients treated with TNFi.^[5–7,19,21–24]^ In addition, Taiwanese RA patients treated with biologics including TNFi had a lower risk of malignancy relative to those treated with DMARDs, as previously reported.^[31]^ The present study further demonstrated a similar and striking difference between etanercept-treated and biologic-naive RA patients. The differences between these 2 reports on Taiwanese individuals may come from their respective study design. First, we used a 1-to-1 matching design and matched patients for MTX use in addition to the duration of RA history, index date of TNFi prescription, and traditional covariates of age and gender. Second, patients who developed cancer in the 1st year of observation were excluded from the present study to prevent misclassification of incident cancer from preexisting cancer. Third, the etanercept-treated group was restricted to patients who used only etanercept. Patients who switched between different TNFi or switched to other biologics because of primary or secondary treatment failure^[33]^ were excluded. Including patients who switch between different biologics may introduce confounding by indication and may cause difficulties in identifying the true effects of TNFi.^[34]^ In contrast, recent report from the British Society for Rheumatology Biologics Registry suggested no additional benefit of etanercept to traditional DMARD in altering the risk of solid cancer in RA patients;^[21]^ however, in the unadjusted model they showed a significant risk reduction by 26% for RA patients treated with etanercept. A trend of risk reduction persisted in the model adjusted by propensity score stratified into deciles, while it was not statistically significant.

Our estimate of cancer risk reduction that is associated with etanercept is close to that reported in a British cohort,^[32]^ but the beneficial effect on the risk of solid cancer demonstrated in our study is in contrast to the negative effect on the risk of lymphoproliferative malignancy in the British cohort. The RA patients treated with etanercept were younger than those treated with DMARDs in the British cohort, whereas age was matched between the cohorts in our study. In addition, the NHI program covers the use of all biologics in Taiwanese patients, and thus the mean disease duration of RA patients to start etanercept was only 2.0 years, and the bias that is due to socio-economic status between patients treated with and without etanercept may not be as prevalent as in the British study.

This study has several limitations. First, although a potential misclassification may have led to an underestimation of the association between TNFi and cancer, the RA patients enrolled in this study were from the Catastrophic Illness Database and met the criteria for RA and malignant diseases. The potential coding error was therefore minimized. Second, there may have been a surveillance bias in patients treated with TNFi, which may have contributed to an increased frequency of cancer in this cohort and thus underestimated the beneficial effect of TNFi on the risk of malignancy. In contrast, a potential selection bias that results from strictly excluding patients who develop cancer in the first year of treatment may have occurred and could over-estimate the beneficial effect of TNFi on the risk of malignancy. However, RA patients who develop cancer before the index date were all excluded, and those TNFi-treated patients who developed cancer during the 1st year of observation (n = 3) were also excluded, along with their matched counterparts, from the analysis. This may preserve the comparability between the etanercept-treated patients and the matched biologic-naive references. Third, as this was not a randomized clinical trial, we cannot attribute all the observed benefit of malignancy reduction to TNFi. The NHIRD does not provide laboratory data or serologic information on inflammation, records of disease activity score, metabolic profiles, body mass index, family history of malignant diseases, or information on personal habits such as cigarette smoking and alcohol drinking, all of which may contribute to cancer risk. In addition, a bias confounded by indication may account for differences in outcomes, that is, those patients who were considered to have a higher risk of cancer did not get treated with TNFi. However, after matching the TNFi-treated and biologic-naive patients for age, gender, RA duration, and MTX use, the prevalence of comorbidities between these 2 groups was generally balanced except peptic ulcer. Fourth, the patients who switched between different TNFi or switched to other biologics were excluded in the present study. Although this method may avoid confounding by indication and help identify the true effect of etanercept, a potential threat to the generalizability of the results should be considered. Fifth, although the TNFi-treated patients may have a more active inflammatory condition and may have a higher risk of developing malignancy than the biologic-naive patients, and although an immortal time bias may have occurred because of early death in patients treated with TNFi, the date of first use of TNFi in the TNFi-treated patients was randomly matched with the biologic-naive patients, and the covariates including comorbidities and concomitant medication were also adjusted in the Cox model. This may allow us to infer causation between TNFi and the risk of cancer occurrence.

Our study demonstrated that the etanercept-treated patients have a more severe inflammatory condition than the biologic-naive patients, as we noted a higher proportion of corticosteroid used in the etanercept-treated patients (80.34%) compared with that in the biologic-naive patients (73.75%), and a persistent higher proportion of MTX use in the etanercept-treated patients. Early death that is due to comorbidity or infection in RA patients treated with TNFi may affect the development of malignancy.^[16,35]^ We adjusted the competing risk that is due to death, while the biologic-treated patients with a high risk of infection are suggested by the NHI guideline to have received adequate treatment before starting TNFi treatment, and those patients should be regularly monitored for the recurrence or new onset of infection. With all these regards, we found significantly fewer malignancy events in patients treated with etanercept relative to those naive to biologics.^[36]^

An association of chronic inflammation with the pathogenesis of cancer^[14,27,29]^ and the role of TNF in cancer development^[12,14]^ have been previously discussed.^[37]^ A profound role for tumor cell-derived TNF-α on malignancy progression has been shown in pancreatic ductal adenocarcinoma and etanercept which has an effect on proliferation and the invasiveness of pancreatic ductal adenocarcinoma was shown as a potentially adjuvant therapy after subtotal pancreatectomy.^[27]^ Infliximab and adalimumab, which are monoclonal antibodies that bind the p55TNF receptor and prevent activation of its receptor,^[29]^ may underlie the lower cancer risk in RA patients who receive TNFi. These results may partially support our finding of a beneficial effect of etanercept on the risk of solid cancer.

The strengths of this study warrant mention. First, the study data source, Taiwan NHIRD, enrolls over 22 million citizens and employees in a national insurance program, a stringent NHI surveillance database.^[1]^ Second, the characteristics of RA patients in the Registry of Catastrophic Illness Database in Taiwan were consistent with previous reports of other populations with RA, with a strong female predominance and a majority of middle-aged individuals.^[1–4]^ These similarities across study populations support the validity of identifying RA patients from the NHIRD. Third, the large sample created adequate study power for subgroup analysis and enabled us to ascertain the impact of TNFi, especially etanercept, on cancer risk in RA patients. Fourth, the sufficiently long period from a mean of 3.9 years and a maximum of 12 years of observation allowed us to compare the effect of TNFi on the development of malignancy.

In an extension of previous findings that treatment with TNFi does not increase the risk of malignancy in RA patients,^[5–7,21–23]^ the current study demonstrated a potential benefit of etanercept on all-cancer^[31,32]^ and solid cancer. Further exploration is warranted to investigate whether other TNFi has a similar benefit.

## Supplementary Material

Supplemental Digital Content
