# Supplementary material for: Reduced risk of all-cancer and solid cancer in Taiwanese patients with rheumatoid arthritis treated with etanercept, a TNF-α inhibitor
Source: Medicine (Baltimore). 2017 Feb 17;96(7):e6055. doi: 10.1097/MD.0000000000006055 (PMC5319502; doi:10.1097/MD.0000000000006055)
Supplement: Supplemental Digital Content [file medi-96-e6055-s001.docx]

*Supplementary Table 1.* Number of all enrolled RA subjects (n = 17,970) with a history of malignancy before the index date of TNFi prescription (n = 47, in total)

| Category of Malignancy | n | % |
| --- | --- | --- |
| Total cancer | 47 | 100 |
| Oropharyngeal cancer | 3 | 6.4 |
| Gastrointestinal cancer | 5 | 10.6 |
| Hepato-pancreato-biliary cancer | 8 | 17.0 |
| Lung and pleural cancer | 7 | 14.9 |
| Skin cancer | 3 | 6.4 |
| Breast cancer | 5 | 10.6 |
| Female genitalia cancer | 6 | 12.8 |
| Male genitalia cancer | 5 | 10.6 |
| Hematological cancer | 5 | 10.6 |
